# ‘Give Up Loving Pop': reimagining oral health promotion through sport, social practice, and prevention

**DOI:** 10.1038/s41415-026-9525-x

**Published:** 2026-02-27

**Authors:** Michael Viggars, Rakhee Patel, Huda Yusuf, Matthew Philpott

**Affiliations:** 41415708757001 Health Equalities Group, 151 Dale Street, Liverpool, L2 2JH, UK; 41415708757002 Faculty of Dentistry, Oral & Craniofacial Sciences, King´s College London, Dental Institute, 16 De Crespigny Park, London, SE5 8AF, UK; 41415708757003 Institute of Dentistry, Queen Mary University of London, Turner Street, London, E1 2AD, UK

## Abstract

Oral diseases such as dental caries remain a significant and inequitable public health challenge among children in England, particularly in socioeconomically deprived communities. Despite their preventable nature, oral health conditions have long been overlooked in national child health strategies and siloed from broader non-communicable disease prevention agendas. This paper presents Give Up Loving Pop (GULP), a pragmatic, school-based oral health intervention delivered in partnership with professional sports clubs and their community organisations. Framed through the lens of health practice theory, GULP moves beyond individualistic models of health education by embedding healthier hydration habits within children's everyday routines, supported by trusted figures such as community sports coaches. Evaluation data from recent local authority delivery projects indicate meaningful shifts in children's drink preferences, particularly in favour of water. This paper situates GULP within the wider context of commercial determinants of health and argues for a more integrated, socially attuned model of oral health promotion; one that aligns with upstream prevention, addresses structural inequalities, and leverages the cultural influence of sport to foster long-term change.

## Key points

- Child oral health inequalities remain both profound and preventable, with free-sugars consumption a major modifiable driver. Yet oral health is routinely peripheral within wider child-health policy, underscoring the need for upstream, setting-based interventions capable of reaching children in communities with the greatest need.
- ‘Give Up Loving Pop' (GULP) leverages the relational authority of professional sports clubs and school environments to reshape hydration practices, integrating social practice theory with structured personal, social, health and economic education delivery. Through material supports (e.g., reusable bottles), routine-focused activities, and credible role models, the programme embeds ‘water-first' norms without relying on moralising or individualistic framings.
- Evaluation across multiple regions demonstrates consistent shifts toward healthier hydration, especially increased water preference in school, sport, and out-of-home contexts, alongside clearer understanding of drink sugar content and modest improvements in oral hygiene routines. These findings indicate GULP's potential as an oral health improvement programme.

## Introduction

Oral health is a fundamental determinant of overall health and has been recognised within NHS England's Framework of Core20PLUS5 approach to reducing health inequalities for children and young people (CYP).^1^ Despite being almost entirely preventable, dental caries continues to exert a substantial burden on CYP, reflecting broader failures in wider public health systems to address the commercial, environmental, and structural determinants of health. One of the main behavioural risk factors of tooth decay is increased consumption of sugar, which is also a risk factor for other chronic diseases.

Mean intakes of free sugars among children aged 4–10 years (12.1% of total energy) and 11–18 years (12.3%) in the UK remain more than double the recommended maximum of no more than 5% of total energy intake.^2^ The scientific evidence is unequivocal: excessive intake of free sugars, particularly through sugar-sweetened beverages (SSBs), are a major contributor to dental caries and erosion,^3^ and other non-communicable diseases, including obesity, type 2 diabetes, and cardiovascular disease.^4^^,^^5^ Oral diseases, often seen through the narrow lens of dental service provision, have been siloed from the wider prevention agendas and continue to receive insufficient strategic focus.^6^

Recent national dental epidemiological data indicate that over one in four five-year-olds (26.9%) continue to experience dental caries in England. The prevalence of tooth decay is higher in the North West, where one in three (36.8%) children experience dental caries.^7^ The burden of oral and dental diseases is closely linked to socioeconomic status, with children living in deprived communities experiencing both higher prevalence and severity of disease.^8^ Dental caries persists as the leading cause of hospital admissions for children aged 5–9 years, with more than 31,000 extractions attributed to caries in 2022–2023 alone, equivalent to 120 operations every working day, and £40.7 million in healthcare costs.^9^ Tooth extractions were more than three times higher in the most deprived areas when compared to the most affluent areas,^9^ highlighting significant oral health inequalities.

The consequences of poor oral health in childhood are wide-ranging and enduring. Children with caries experience are more likely to have diminished academic attainment compared to children with no caries experience,^10^ and have increased school absenteeism.^11^ Evidence also links untreated caries and associated pain to impaired sleep^12^ and fewer sleeping hours appears to be an independent risk factor for dental caries.^13^ Children with dental caries are significantly less likely to be school-ready at age five, with this association persisting independently of socioeconomic status.^14^ Oral health, therefore, cannot be viewed in isolation. It is intrinsically linked with the wider determinants of health with impacts on educational, social, and health inequalities.

The introduction of the UK Soft Drinks Industry Levy in 2018 demonstrated the potential for upstream fiscal measures to reduce sugar consumption and contributed to declines in hospital admissions for tooth extractions.^15^^,^^16^ However, slow progress on restricting advertising of high fat, sugar, and salt products to children^17^ highlights ongoing policy inertia and risks maintaining the obesogenic and cariogenic environments in which our children are growing up.

While recent national policy has reaffirmed the importance of supervised toothbrushing schemes as a cost-effective intervention for improving child oral health, these programmes on their own^18^^,^^19^ do not address the wider social determinants of health, nor tackle poor nutrition and pervasive SSB consumption, highlighting the need for innovative common risk factor approaches that integrate oral health intro wider public health, education and social policies.

## Sport as a tool for more equitable health promotion

To make a real difference, oral health promotion must extend beyond dental practices and tackle the social determinants of health. Sport occupies a distinctive position within the public sphere, characterised by high levels of cultural capital, social legitimacy, and sustained engagement.^20^ When harnessed effectively, sport offers a relational and socially embedded space to influence health-related behaviours at scale.

Through the power and pull of professional sports clubs, their club community organisations (CCOs)^21^ can leverage credibility, visibility, and aspirational value on health promotion initiatives in a manner that conventional health and education systems may not easily replicate. The role of CCOs and coaches in delivering health and education interventions has become increasingly mainstreamed across the UK over the last decade.^22^ Initially established to boost sports participation, CCOs have evolved into trusted delivery agents for school-based and public health programmes addressing physical health, mental wellbeing and social inclusion.^23^

Frequently perceived by CYP as relatable, trustworthy, and motivational figures,^24^ community sports coaches possess a unique capacity to facilitate change, not through formal authority, but through ongoing relationships grounded in routine contact and shared purpose. When these coaches deliver school-based interventions such as ‘Give Up Loving Pop' (GULP), the health messages are imbued with the authenticity and social resonance of the club's wider identity, enhancing both engagement and receptivity.^22^ Catalysed by the Government's Sporting Future,^25^ this shift reflects a broader realignment of sport policy toward public health outcomes. As such, embedding oral health promotion within sport-affiliated programmes represents a scalable and socially attuned strategy for addressing entrenched inequalities in oral health outcomes, particularly when products such as sports drinks are often consumed in non-sports settings.^26^

This article aims to situate and critically examine the GULP intervention within the broader context of child oral health inequalities and health promotion through sport. It seeks to outline the development and theoretical framing of GULP, describe its implementation across community sport settings, and present preliminary findings on changes in children's drink preferences.

## Methods

### Programme development

**Fig. 1:**
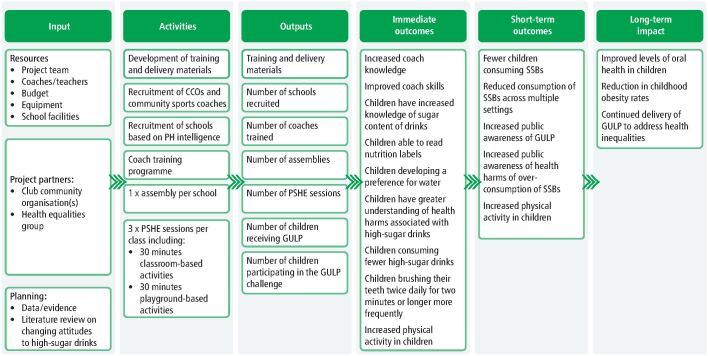
Logic model for the GULP intervention

A school-based personal, social, health and economic education intervention for children aged 7–11 years (KS2) was co-developed in Northwest England to reduce SSB consumption and address unhealthy weight and tooth decay. The intervention's theory of change is outlined in Figure 1.

### Phase 1: social marketing campaign

In the early 2010s, there was a growing concern around the proliferation and normalisation of consumption of SSBs among CYP. In response, the Northwest directors of public health, through a nascent programme delivered by a health equalities group (HEG) called Food Active, highlighted the need for a campaign to raise awareness of the health harms of overconsumption of SSBs and to begin lobbying the government to introduce a sugar tax. Launched in 2015, GULP was conceived as a social marketing and advocacy campaign. The campaign also recognised social media's potential to amplify messaging and to serve as a platform for ongoing dialogue, relationship-building, and audience engagement.^27^

GULP sought to position young people and their communities as active participants in the growing discourse around SSB consumptions and associated health harms and to critically interrogate the aggressive and often deceptive marketing strategies employed by the soft drinks industry, particularly those targeting children and communities experiencing disadvantage.

### Phase 2: development of KS2 programme

The public health team in Blackpool Council, part of the Food Active network, had seen the potential for their local football club, Blackpool FC, through their CCO, Blackpool Football Club Community Trust, to deliver evidence-based health education on SSBs in primary schools. The rationale was to instil and support healthier habits around hydration at a critical juncture in children's development between reception and Year 6 where data from the National Child Measurement Programme consistently demonstrates that the prevalence of obesity doubles in this time.^28^

Taking learning from the intervention in Blackpool and other novel school-based interventions,^29^ GULP was developed into a structured programme, delivered over four weeks with interactive lessons, a physical activity component and a guided challenge (Fig. 1).

The content of the intervention included children learning about the harms of SSBs on oral health and the value of good oral hygiene practices; how to read and interpret nutrition labels; and the importance of hydration to support educational attainment and performance in sport and physical activity. Latterly, environmental messaging on plastic pollution was integrated, reflecting growing concern among CYP about its environmental impact.^30^ This refined programme was first piloted by staff from HEG in a school in Liverpool, before community sports coaches from Everton in the Community (the charitable arm of Everton Football Club) were trained to deliver the first project across schools in Sefton in 2016.

Subsequently, GULP has been commissioned by local authority public health teams and NHS organisations to enhance upstream, school-based prevention, aligning with evidence demonstrating that schools are critical settings for health promotion and that interventions targeting SSB consumption and physical activity can support meaningful behaviour change.^31^ Subsequently, GULP has been commissioned by local authority public health teams and NHS organisations seeking to strengthen upstream, school-based prevention. HEG acts as the coordinating organisation, supporting commissioners to design projects, identify delivery partners, and build capacity within CCOs to deliver GULP, with commissioners identifying primary schools serving populations at greatest risk of dental decay and excess weight. Since 2016, over a dozen CCOs have delivered the GULP programme to over 11,000 children across England.

### Participants and recruitment of schools

During the 2023/2024 academic year, five CCOs were commissioned to engage and recruit primary schools across Lancashire and Blackburn with Darwen. The programme targeted Year 3 pupils (aged 7–8 years), with delivery aiming to focus on schools serving communities experiencing the greatest oral health inequalities.

Participating schools were identified through collaboration between CCOs and local authority public health teams, drawing on the Index of Multiple Deprivation to prioritise those located within the most deprived 30% of neighbourhoods nationally. This targeted approach was consistent with the strategic objectives of both Lancashire County Council and Blackburn with Darwen Borough Council^32^ to reduce health inequalities in SSB consumption, oral health, and childhood obesity.

### Measures, data and analysis

The primary outcome was change in children's consumption of SSBs and preference for water across a range of settings. Secondary outcomes included knowledge of the health harms of SSBs, understanding of the sugar content of common drinks, and awareness of oral health. All outcomes were assessed through pre- and post-programme online surveys administered in schools, capturing self-reported beverage preferences across school, sport, and eating-out settings, alongside oral hygiene behaviours. Instruments were age-appropriate for KS2 pupils (7–8 years) and based on previous GULP evaluations. Anonymised raw survey data underpinning all analyses are available in Supplementary File 1.

Descriptive data were analysed to assess changes in knowledge and behaviours numbers and proportions. To evaluate the impact of the GULP intervention, changes in children's drink preferences were assessed using paired data and *McNemar's test* across three everyday settings: school, sport, and out-of-home.

### Preliminary results

Across the delivery period, a total of 89 schools participated, encompassing 141 Year 3 form classes. Of these, 62% of schools were situated within the 30% most deprived wards nationally, reflecting a clear equity focus on programme recruitment and delivery (Table 1).

**Table 1:** Participating schools by deprivation across Lancashire and Blackburn with Darwen

**Local authority**	**Number of schools engaged**	**Number of form classes receiving GULP**	**Percentage of schools in 30% most deprived wards**
Lancashire	66	104	63%
Blackburn with Darwen	23	37	62%
**Total**	**89**	**141**	**62%**

From the approximately 4,230 children who received GULP in 2023/24, 813 paired datasets were received for analysis (Table 2). Instances of missing data were attributable to limited teacher participation in survey administration and routine pupil absences (e.g., illness).

**Table 2:** Participating children and data submitted

**Local authority**	**Approximate number of children engaged**	**Pre-surveys completed** **Response rate (%)**	**Post-surveys completed** **Response rate (%)**	**Paired datasets** **Response rate (%)**
Lancashire	3,120	726 23.3%	661 21.2%	564 18.1%
Blackburn with Darwen	1,110	622 56.0%	488 44.0%	249 22.4%
**Total**	**4,230**	**1,348** **31.9%**	**1,149** **27.2%**	**813** **19.2%**

Evaluation data from two local delivery projects during the 2023/24 school year provide evidence of GULP's initial impact. While data were aggregated for analysis, preliminary comparisons indicated consistent patterns across both sites.

Significant positive shifts in beverage preferences were observed across all three settings (Table 3). The proportion of children selecting water increased from 50.2% to 65.1% at school, from 40.6% to 57.1% during sport, and from 18.1% to 30.3% in out-of-home contexts. McNemar's *χ*^2^*tests* confirmed these changes were statistically significant in each setting (*p* <0.001). The largest relative gains were observed in school and sport environments, suggesting that structured and health-promoting settings may be most conducive to reinforcing water-first behaviours.

**Table 3:** Summary of drink preference changes among children following the GULP intervention, by setting

**Setting**	**n (paired)**	**Pre-intervention preference for water**	**Post-intervention preference for water**	**Change in preference between pre- and post-intervention**	**McNemar χ²**
School	813	50.2%	65.1%	14.9%	39.02*
Sport	813	40.6%	57.1%	16.5%	46.31*
Out-of-home	564	18.1%	30.3%	12.2%	22.78*
*p <0.001. Contingency tables and McNemar's *χ²* test results showing changes in children's selection of water versus other drinks before and after the GULP intervention, across school, sport, and out-of-home settings. Data reflect paired responses only. Statistically significant shifts were observed in all settings, with absolute increases in water preference ranging from 12.2 to 16.5 percentage points (all *p* <0.001)					

## Discussion

Initial findings suggest that GULP is both deliverable and effective across varied local authority settings, and capable of supporting meaningful shifts in hydration practices among children, particularly those at greater risk of poor oral health. While the evaluation was uncontrolled and based on self-report data, the consistency and statistical strength of the findings reinforce the programme's potential.

GULP can be critically understood through the lens of health practices, offering a deliberate departure from traditional, individualistic models of health behaviour that have long dominated public health research and policy.^33^ Rather than focusing on individual decision-making or rational knowledge deficits, GULP foregrounds the material and social contexts in which drinking practices are embedded. Just as twice-daily toothbrushing is encouraged until it becomes an automatic part of a child's day, GULP seeks to position drinking water as the default practice during school, play and in out-of-home food settings, rather than an explicit ‘healthy choice' made against a backdrop of unhealthy options. This reframes hydration not as a cognitively deliberated behaviour grounded in abstract nutritional knowledge, but as an embodied, relational and socially shared practice that is accessible and socially sanctioned.

Crucially, GULP avoids the moralising and cognitive burden often associated with conventional health education, which demands individual vigilance across a wide range of dietary products, many of which children have no purchasing control over.^34^ Instead, the intervention works with children's limited agency, offering a practice they can adopt with consistency and confidence. In this way, GULP reflects a shift from targeting individual behaviours to intervening in the ‘lives' of social practices themselves,^35^ disrupting unhealthy routines not through information alone, but by transforming the elements that constitute them: materials, competencies, and meanings.

In alignment with these theoretical shifts, GULP does not aim to isolate or correct discrete ‘bad choices' but rather to cultivate new configurations of practice, making water consumption a normative, rewarding and structurally supported activity. It exemplifies a more relational, sociologically attuned model of intervention that acknowledges that what children do is not merely the product of conscious choice, but of situated, embodied, and collectively reproduced practices.^33^^,^^35^

Several limitations should be noted. The programme was not part of a formal research study, and a pragmatic pre–post design was used to enable delivery under real-world conditions in which randomisation was not feasible. The measures used in this preliminary analysis require refinement to ensure the inclusion of validated tools that strengthen data quality. Assessment of longer-term impacts would also benefit from incorporating clinical indicators such as dental decay experience and body mass index. A more comprehensive analytical approach could account for age, sex, deprivation, and ethnicity. To support wider scalability and sustainability, interventions such as GULP would benefit from a more formal and consistent evaluation framework that integrates robust outcome measurement, implementation fidelity, and longitudinal assessment, offering credible evidence to guide commissioning, policy development, and engagement with underserved populations.

### Challenges and recommendations for policy and practice

Leveraging professional sport for public health presents important opportunities but also structural challenges. From a commercial determinants perspective,^36^ sponsorship by unhealthy commodity industries, including soft drink companies, is widespread across professional sport,^37^ creating inherent tensions when CCOs are asked to deliver GULP. These tensions are intensified where club governance structures prioritise maintaining favourable relationships with commercial partners whose interests may conflict with public health aims.

Professional football clubs also exert substantial cultural influence on children and young people, shaping brand loyalties and consumption patterns.^38^ In this context, mixed messaging arises when clubs promote GULP's water-focused message while simultaneously endorsing SSB brands. Such contradictions risk diluting the intervention's credibility, reframing water as a neutral choice rather than a challenge to the normalisation of SSBs within sport and wider society. Consequently, the potential for GULP to shift norms and displace unhealthy consumption behaviours may be curtailed when health objectives appear misaligned with the commercial priorities of the sporting institution.

Considering the abolition of NHS England^39^ and the directive for integrated care boards to achieve substantial operational savings,^40^ rebalancing investment toward upstream, population-level prevention will be challenging. However, oral health must be repositioned as a core component of integrated child health strategies that address shared risk factors; particularly the consumption of SSBs. The continued burden of dental disease and obesity, both of which disproportionately affect children in deprived communities, is strongly linked to the widespread availability and marketing of unhealthy commodities. In this context, interventions such as GULP offer a tangible example of how oral health promotion can be effectively embedded within broader public health agendas.

## Conclusion

The continued burden of oral disease among children, particularly in deprived communities, demands a more ambitious and integrated approach to prevention. GULP demonstrates how oral health promotion can move beyond traditional paradigms of behaviour change and siloed dental education, towards interventions that embed healthy practices into the social fabric of children's daily lives. By operationalising health practice theory within trusted sport and education settings, GULP reframes hydration not as a matter of individual willpower, but as a shared, socially sanctioned routine. In doing so, it challenges the structural and commercial forces that normalise sugar consumption, while avoiding the moralising tones that often characterise public health messaging and school-based programmes. When decoupled from unhealthy commodity partnerships, sport offers a culturally resonant platform for scalable prevention.

Interventions such as GULP illustrate how oral health can be positioned as a core component of upstream child health policy, aligned with wider efforts to reduce non-communicable diseases and health inequalities. Future strategies must build on this by recognising and investing in the environments that shape children's daily routines, representing not just a strategic shift but a necessary reimagining of effective, equitable, and practice-oriented oral health promotion.

## Data Availability

The data generated and analysed for this study are not publicly available, as participating schools and children were not informed that their data would be archived or shared beyond the purposes of programme evaluation. Researchers seeking access to anonymised data for non-commercial research may submit a request to the corresponding author.
